# Characterisation of the Novel Filamentous Phage PMBT54 Infecting the Milk Spoilage Bacteria *Pseudomonas carnis* and *Pseudomonas lactis*

**DOI:** 10.3390/v15091781

**Published:** 2023-08-22

**Authors:** Frank Hille, Stefanie Gieschler, Erik Brinks, Charles M. A. P. Franz

**Affiliations:** Department of Microbiology and Biotechnology, Max Rubner-Institut, Federal Research Institute of Nutrition and Food, Hermann-Weigmann-Str. 1, 24103 Kiel, Germany; stefanie.gieschler@mri.bund.de (S.G.); erik.brinks@mri.bund.de (E.B.); charles.franz@mri.bund.de (C.M.A.P.F.)

**Keywords:** filamentous phage, milk spoilage, biofilm, *Pseudomonas*, *Inoviridae*

## Abstract

Filamentous bacteriophages are lysogenic and pseudo-lysogenic viruses that do not lyse their host but are often continuously secreted from the infected cell. They belong to the order *Tubulavirales*, which encompasses three families, with the *Inoviridae* being the largest. While the number of identified inoviral sequences has greatly increased in recent years due to metagenomic studies, morphological and physiological characterisation is still restricted to only a few members of the filamentous phages. Here, we describe the novel filamentous phage PMBT54, which infects the spoilage-relevant *Pseudomonas* species *P. carnis* and *P. lactis*. Its genome is 7320 bp in size, has a mol% GC content of 48.37, and codes for 13 open-reading frames, two of which are located on the (−) strand. The virion exhibits a typical filamentous morphology and is secreted from the host cell at various lengths. The phage was shown to promote biofilm formation in both host strains and, therefore, has potential implications for milk spoilage, as biofilms are a major concern in the dairy industry.

## 1. Introduction

Filamentous phages from the family *Inoviridae* are long and thin filaments (6–8 nm in diameter and 800–2000 nm in length) that contain a circular ssDNA genome [1,2,3,4]. The phage filament itself is composed of several thousand subunits of the phage’s major coat protein, which are usually between 44 and 86 amino acid residues in length and are arranged in a helical array around the ssDNA core [4,5]. The ends of the filaments, on the other hand, consist of only a few copies of minor coat proteins. The size of the phage filament is often a function of the size of the genome, which ranges from 4 to 12 kbp and which encodes 4 to 17 or more genes [2,4].

Filamentous phages infect Gram-negative and Gram-positive bacteria, as well as archaea [6]. These phages have been described as parasites, as they exist at the expense of the bacterial host without killing it [7]. Others have described them as an example of microbial sharing ecology, postulating that filamentous phages impose very little burden on the host bacteria and compensate for this by either promoting biofilm formation, providing toxins and/or other factors that increase virulence, or modifying behaviour that provides novel motile activity to their bacterial hosts [8]. In contrast to lytic bacteriophages, the filamentous phages are assembled in the host’s membrane and extruded across the cellular envelope without lysing the host cell, i.e., while the bacterium continues to grow [4,7,8]. Prior to egress, filamentous phages replicate either pseudo-lysogenically as episomes or integrate into the host genome. The latter is accomplished either by host-mediated site-specific recombination using the XerC/XerD machinery, or by phage-encoded recombinases [8,9].

Filamentous phages infecting a range of Gram-negative bacteria have been described, including the well-studied *E. coli* Ff (M13, f1 and fd), Ike, and If1 phages [10] and the Pseudomonas phage Pf1 [11]. Phage Pf1 was first described by Takeya and Amako, 1966 [12]. Further *P. aeruginosa* Pf phages were subsequently isolated, and these “Pf1-like” phages Pf1, Pf4, Pf5 Pf6, and Pf7 are thought to be strain-specific variants of an ancestral prophage, with their specificity being due to strain-specific variation of the type IV pili receptors [4,8,13].

In this study, we describe the filamentous phage PMBT54, which was isolated from raw milk and infected one strain each of the *Pseudomonas* species *P. carnis* and *P. lactis*. Both *Pseudomonas* species are closely related [14], and the originally described type strains were isolated from food industrial environments (meat [14] and raw milk [15], respectively) and are considered to be spoilage-relevant members of their genus. *P. lactis* possesses especially high spoilage potential due to the production of the heat-stable protease AprX, which can withstand heat sterilisation procedures and negatively impact the shelf life of milk products [16]. The specific strains *P. carnis* M132 and *P. lactis* L1-92 used in this study were both isolated from raw milk (Supplementary Table S1).

The aim of the investigation was to isolate and identify bacteriophages with activity against *Pseudomonas* spp. involved in milk spoilage in order to assess their potential for biopreservation. While filamentous phages are generally not suitable for biocontrol applications because they do not lyse their hosts, the presence of those phages in the dairy environment might have other implications, as they can provide certain physiological advantages to their host, such as biofilm formation. Therefore, we analysed the morphology and genetics of the phage, as well as physiological characteristics involving its host strains.

## 2. Materials and Methods

### 2.1. Phage Isolation and Growth Conditions

The *Pseudomonas* species used in this study are listed in Supplementary Table S1. If not stated otherwise, all strains were grown in Caso broth at 30 °C under static conditions. To screen for phages infecting these strains, we analysed raw milk samples provided by a dairy plant in southern Germany.

Raw milk samples were mixed with 10% lactic acid until a pH between 4.2 and 4.6 was reached, and then they were incubated at room temperature for 5 min to allow milk proteins to precipitate. After centrifugation (20 min, 4 °C, 17,000× *g*), the supernatant (whey) was filtered through a 0.45 µm filter; 0.2 mL of the processed milk sample was then mixed with 0.1 mL of an overnight culture of each *Pseudomonas* strain, incubated at room temperature for 10 min, and further incubated overnight at 25 °C after adding 5 mL of fresh Caso broth. The next day, the cultures were centrifuged (10 min, room temperature, 6000× *g*) and filtered through a 0.45 µm filter; 200 µL of the filtrate was spotted on Caso soft agar plates supplemented with 10 mM MgCl_2_, 10 mM CaCl_2_, and 0.25% glycine and containing the respective host strain. If a lysis zone appeared after overnight incubation, it was scraped off and soaked in SM buffer (5.8 g/L NaCl, 2.5 g/L MgSO_4_ × 7 H_2_O, 2.4 g/L Tris, pH 7.4) for 2 h to allow the phages to diffuse out of the agar. After filtration (0.45 µm), 200 µL of the phage suspension was spotted again as described above. Next, the spot was scraped off, soaked in SM buffer for 2 h, and filtered (0.45 µm). The suspension was subsequently diluted in quarter-strength Ringer’s solution, and various dilutions were plated on the host bacterium as described above to yield single plaques. A single plaque was scraped off, soaked in SM buffer as described above and, after filtration (0.45 µm), the suspension was plated again and single plaques were isolated two more times to ensure purity.

### 2.2. Host Infection, DNA Extraction, and Sequencing

The double-stranded episomal variant of the phage genome was used for sequencing. For this, 0.1 mL of phage lysate was mixed with 0.1 mL of 40 mM CaCl_2_, after which 0.3 mL of an overnight culture of *P. carnis* M132 was added. The suspension was filled up to 5 mL with fresh Caso broth and incubated overnight at 25 °C. Next, the infected cells were pelleted at 6000× *g* for 10 min, and the phage genome was extracted using the Plasmid Mini AX purification kit (A&A Biotechnology, Gdansk, Poland). The extraction yielded plasmids present in the host as well as the phage genome and was further subjected to Illumina sequencing.

For fragmentation of the DNA, the Covaris M220 ultrasonicator (Covaris, Woburn, MA, USA) with microtube-50 AFA Fiber Screw-Caps was used to yield a fragment length of 400 bp. The library was prepared using the TruSeq Nano DNA LT Library Preparation Kit (Illumina, San Diego, CA, USA) according to the manufacturer’s instructions. Sequencing was performed on an Illumina MiSeq sequencer (Illumina, San Diego, CA, USA) with 2 × 251 cycles using the MiSeq Reagent Kit v2 (500 cycles).

### 2.3. Sequence Analysis and Taxonomic Classification

Sequence reads were assembled using SPAdes (v3.15.5) in metaviral mode, and the assembled contigs with a coverage > 10.0 were subjected to a Blastn search [17]. Three contigs matched to plasmid sequences present in various *Pseudomonas* species (see Supplementary Table S2) that could be explained by the plasmid DNA extraction procedure used. One contig showed high similarity to a metagenome-assembled genome derived from a filamentous phage labelled ctbd3 (Accession: MH616883) and was thus further analysed.

For gene calling and annotation, the viral contig was analysed using RASTtk [18]. In order to exclude the possibility that the assembly-generated artificial start of the sequence of the circular genome could influence gene calling by being located within a coding sequence, we repeated the process with an alternative starting location and curated the final annotation manually. For this, open-reading frames that were detected using both starting locations were annotated as true ORFs. All detected ORFs were further analysed with HHsuite [19] against the Pfam, PDB, and RefSeq databases to predict further ORF products. For ambiguous ORFs (i.e., those that were not identical between the described annotation procedures but spanned a similar location on the contig), we annotated them as true ORFs when they showed a significant hit in the HHsuite analysis (Supplementary Table S3). Hits with a probability above 80% and coverage above 80% were considered significant. When multiple hits were detected, those that matched a phage/inovirus protein or that were derived from a PDB entry were favoured.

Taxonomic analyses were conducted using VipTree [20], which uses a genome-wide tBlastx approach to identify closely related species in the VirusHost database (RefSeq release 215; [21]). The genome sequences of PMBT54 and ctbd3 were uploaded to the VipTree server and analysed against all single-stranded DNA viruses present in the database. Hits with an all-against-all genomic similarity score (S_G_) above zero were used to build a proteomic tree.

Direct genome comparisons between PMBT54, ctbd3, and Pseudomonas phage Pf3 were generated using Easyfig [22]. Blast files were generated using the tBlastx approach, with the minimum identity value set to 40%.

The gene-sharing network analysis was conducted using vContact2 v0.11.3 [23]. For this, all metagenomic inoviral sequences identified in [6] were downloaded as GenBank files and coding sequences were extracted using a custom script (Supplementary Table S3) in Python v3.10 [24] and the package Biopython v1.79 [25]. The coding sequences identified for PMBT54 were included, and the gene-sharing network was generated using standard settings without a reference database. The network was visualised using Cytoscape v3.9.1 [26].

### 2.4. Phage Growth Curve

An overnight culture of *P. carnis* M132 was diluted 1:50 in fresh Caso broth and incubated with agitation (150 rpm) at 25 °C until an OD (620 nm) of approximately 0.1 was reached. The culture was centrifuged for 10 min at 3000× *g* and resuspended in fresh Caso broth. Next, the bacterial cell number was adjusted to 1 × 10^7^ CFU/mL in a new flask containing 10 mM CaCl_2_, 10 mM MgCl_2_, and 1 × 10^6^ PFU/mL of phage PMBT54 (MOI = 0.1), for a final volume of 10 mL, and incubated at 25 °C with agitation (150 rpm). The phage titre was determined directly upon addition of the culture (timepoint zero) and at regular time intervals. For this, 1:10 serial dilutions in SM buffer were prepared, and appropriate dilutions were plated on a separate culture of the host strain. The plates were incubated at 25 °C overnight, and the number of plaques was used to calculate the phage titre in PFU/mL. Similarly, the growth behaviour of the hosts (infected and uninfected) was assessed, and CFU/mL values were determined at certain timepoints post-infection by plating appropriate culture dilutions.

### 2.5. Host Spectrum

The host spectrum of phage PMBT54 was tested for the *Pseudomonas* species stated in Supplementary Table S1. For this, 10 µL of the phage suspension (propagated on the isolation strain *P. carnis* M132) was spotted on a Caso agar plate containing the respective strain. If a lysis zone was visible after overnight incubation at 25 °C, the efficiency of plating (EOP) was determined for these strains. For this, the titre of the phage was determined on an overnight culture of the respective host strain by plaque assay, as described above, where the highest observed titre equalled an infection efficiency of 100% (EOP = 1). Lower titres were set in relation to the highest obtained titre.

### 2.6. Biofilm Formation Assay

To assess the effects of phage PMBT54 on the biofilm formation of *P. carnis* M132 and *P. lactis* L1-92, we conducted a modified version of the microtiter plate assay described by [27]. For this, both strains were grown overnight in Caso broth at 30 °C. The cultures were split, centrifuged at 6000× *g* for 5 min, and resuspended in fresh Caso broth and 50% milk medium (50% UHT milk with 3.5% fat and 50% quarter-strength Ringer’s solution), respectively. In a total volume of 100 µL of the respective media, the cell number was adjusted to 1 × 10^6^ cells/mL and mixed with 1 × 10^6^ PFU/mL of the phage PMBT54 (MOI = 1). For the uninfected control, a Caso broth and 50% milk medium was used instead of the phage suspension. Blanks contained only media. Samples were statically incubated in 96-well flat-bottomed microtiter plates at 25 °C for 2 days.

After incubation, planktonic cells were removed by pipetting and shaking out the liquid and washing each well of the microtiter plate twice with double-distilled water. To kill and fix cells in the biofilm, 200 μL ≥ 99.9% methanol per well was added and incubated for 15 min at room temperature. After washing twice with water, 125 µL of a 0.5% solution of crystal violet was added and incubated for 15 min at room temperature. Wells were rinsed 3 times with water as described above. The plate was dried for 2 h before quantification.

For quantification, 125 µL of 30% acetic acid was added to the stained wells and incubated for 15 min at room temperature to solubilise the crystal violet before transferring 100 µL to a new microtiter dish for absorbance measurements at 550 nm with a SPARK^®^ multimode microplate reader (Tecan Group Ltd., Männedorf, Switzerland).

### 2.7. Transmission Electron Microscopy (TEM)

Phage samples were prepared by infecting *P. carnis* M132 as described above. After overnight growth of the infected host, the culture was centrifuged (10 min, 3000× *g*), and the supernatant was filtered (0.45 µm) and diluted 1:100 in SM buffer before TEM imaging.

For visualisation of an infected host, an overnight culture of *P. carnis* M132 was adjusted to 5 × 10^7^ CFU/mL in fresh Caso broth in a flask containing 10 mM CaCl_2_, 10 mM MgCl_2_, and 5 × 10^7^ PFU/mL of phage PMBT54 (MOI = 1) in a final volume of 5 mL, before being incubated at 25 °C with agitation (150 rpm) for 2 h. Next, the culture was centrifuged (3000× *g*, 10 min) and the supernatant was discarded to remove free phages. After another hour of incubation under the same conditions, the samples were subjected to TEM imaging. Uninfected controls were treated identically, except SM buffer was added instead of the phage.

For transmission electron microscopy, phage samples were absorbed on carbon films for 20 min, and bacterial samples (infected und uninfected) were additionally fixated with 1% glutaraldehyde for 10 min. The carbon films were washed twice with deionised water and stained for a few seconds with 1% uranyl acetate. Electron micrographs were taken on a Talos L120C transmission electron microscope (Thermo Fisher Scientific, Eindhoven, the Netherlands) using a 4 k × 4 k Ceta camera (Thermo Fisher Scientific, Eindhoven, the Netherlands) set to an acceleration voltage of 80 kV.

## 3. Results and Discussion

### 3.1. Phage Identification and Morphological Characterisation

PMBT54 was isolated from raw milk provided by a dairy plant from southern Germany. The phage formed turbid plaques on the *P. carnis* M132 host strain lawns used for isolation. Single plaques were purified three times, and phage purity was visually assessed by TEM analysis.

**Figure 1:**
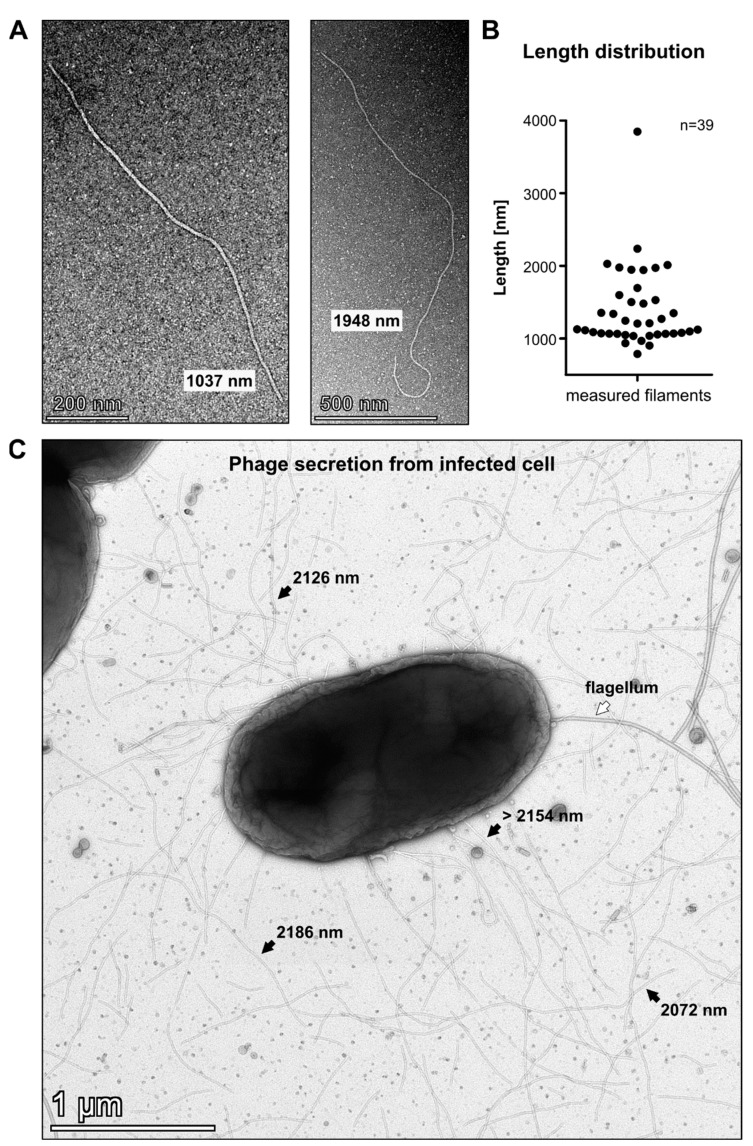
Morphology of PMBT54: (**A**) TEM image of purified phage particles. Various filament lengths were observed. (**B**) Length distribution of 39 measured filaments from purified phage solution. (**C**) TEM image of secreted phage virions from an infected *P. carnis* M132 cell. Black arrows indicate the lengths of large phage filaments. White arrow indicates a single flagellum of the host cell.

For morphological characterisation, the phage was propagated on the host strain used for isolation, and the filtered lysate was subjected to TEM analysis. PMBT54 displayed a filamentous virion morphology, indicating that this phage belongs to the *Tubulavirales* order encompassing the filamentous phages of the families *Inoviridae*, *Paulinoviridae*, and *Plectoviridae,* according to the latest ICTV classification. Interestingly, the filament length was variable and ranged from roughly 1000 nm to approximately 4000 nm (Figure 1A,B). Upon measuring the lengths of 39 clearly distinguishable filaments, we found that the majority displayed a length of around 1000 nm, while there was another cluster of around 2000 nm long filaments (Figure 1B). This could indicate that some of the filaments physically broke during sample preparation, which could have possibly led to the observed differences in filament lengths.

To obtain a better understanding of the various filament lengths, we investigated phage secretion by conducting TEM analysis of an infected host (Figure 1C and Supplementary Figure S1). Among smaller filaments, we found multiple virions that were still attached to the host and displayed lengths above 2000 nm. This suggests that, indeed, the virions exhibit a rather large morphology, and that smaller filaments might have been damaged during sample preparation. Alternatively, virions might be secreted from the host at various lengths and potentially harbour more than one genome copy per virion. In the well-studied *E. coli* Ff filamentous phages, assembly termination requires the capsid protein pIII, which, if deleted, causes the host to produce long, non-infectious filaments that are not released from the host cell and contain multiple copies of the phage genome [28]. Interestingly, if pIII is supplied in trans in cells infected with the pIII deletion mutant, the filament length is directly dependent on the intracellular pIII concentration. High intracellular pIII concentrations produce filaments that display the same length as the wt and contain one genome copy. Lower pIII concentrations yielded longer filaments that contained multiple genome copies. These filaments were still able to be secreted from the cell and remained infectious in contrast to cells that completely lacked pIII expression [28]. Considering those findings, it is possible that a similar mechanism is responsible for the observed differences in PMBT54 filament length: the protein controlling assembly termination might not be expressed in sufficient amounts and, thus, may cause the release of filaments that vary in length. Even though physical damage to some virions certainly contributed to the detected variety in filament length, the observation of two length clusters at 1000 and 2000 nm, which might represent virions containing one and two genomes, respectively, rather suggests a biological explanation. However, whether this is the natural egress mechanism of PMBT54, or whether other reasons like suboptimal protein expression in the host *P. carnis* M132 affect assembly, remains to be determined.

Filamentous phages typically utilise host pili as a primary receptor for infection, while various secondary receptors located on the cell surface have been identified for different filamentous phages as well [8]. However, the primary host of PMBT54 identified in this study, *P. carnis* M132, seems to lack any detectable pili. TEM analysis revealed the expression of flagella by the bacterium, but no pili could be observed (Supplementary Figure S1), indicating that PMBT54 might not depend on host pili for attachment. Further experiments are therefore required to determine the receptor for the phage PMBT54 in this host strain.

### 3.2. Physiological Characterisation

**Figure 2:**
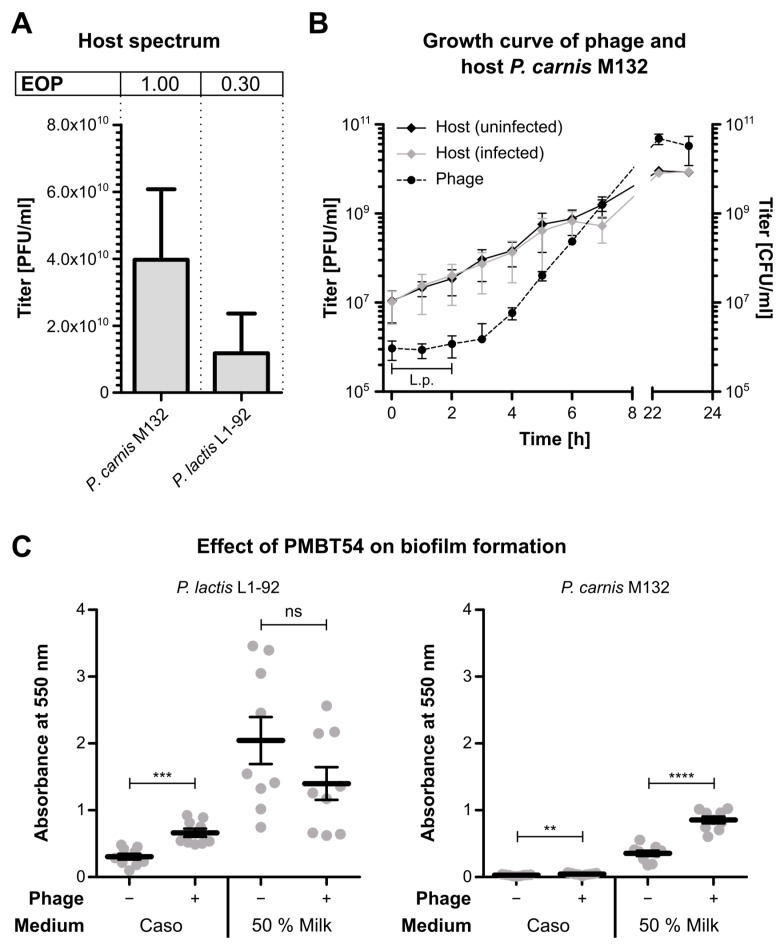
Physiological analysis of PMBT54: (**A**) Efficiency of plating (EOP) of phage PMBT54 on susceptible *Pseudomonas* strains. Preliminary spot tests were conducted on 27 *Pseudomonas* strains (see Table S1) to test host susceptibility. The EOPs of the phage (propagated on *P. carnis* M132) were subsequently determined on overnight cultures of the two identified susceptible strains, *P. lactis* L1-92 and *P. carnis* M132. Error bars indicate the standard deviation of five biological replicates. (**B**) Growth curve of PMBT54 on isolation strain *P. carnis* M132 and growth curves of *P. carnis* M132 (infected and uninfected). The host strain was infected at a MOI of 0.1, and both phage and bacterial titres were measured at regular intervals for 7 h post-infection and after overnight incubation. Error bars indicate the standard deviation of three biological replicates. L.p. = latent period. (**C**) Biofilm formation of uninfected and infected host bacteria in Caso broth and 50% milk medium. Biofilm formation was quantified by crystal violet staining and absorbance measurement at 550 nm. Data points of three biological replicates, each encompassing three technical replicates, are illustrated as dots. Bars represent the mean of all points, including standard deviation. Statistical significance was assessed by calculating the *p*-values of two indicated samples using an unpaired *t*-test; ns = *p* ≥ 0.05 (not significant); ** = *p* ≤ 0.01; *** = *p* ≤ 0.001; **** = *p* ≤ 0.0001.

The host range of PMBT54 was tested against 27 milk-spoilage-relevant *Pseudomonas* strains (Supplementary Table S1) by conducting spot assays (not shown). In addition to the strain *P. carnis* M132 used for isolation, spot formation was only observed for *P. lactis* L1-92. Next, the efficiency of plating (EOP) was determined for both strains and revealed that PMBT54 infected *P. lactis* considerably less efficiently compared to *P. carnis* (EOP = 0.30, Figure 2A). It is noteworthy that we conducted the EOP experiments with phage lysates propagated on the isolation strain *P. carnis* M132. Hence, we cannot exclude the possibility of potential adaptive processes resulting in a higher EOP value for this strain. It has been shown that infection efficiencies can be dynamic and adaptable, depending on the host used for propagation [29], but this was not further explored in the present study.

To better understand the infection cycle of PMBT54 in the *P. carnis* host strain, we conducted a growth curve experiment. For this, the host was infected at an MOI of 0.1, and the phage titre in the supernatant was determined at regular intervals (Figure 2B). The virion numbers started to increase between 2 and 3 h post-infection, suggesting that PMBT54 displays a relatively long latency period. TEM analyses supported these findings, as no phage particles could be detected 90 min post-infection (Supplementary Figure S1). After 120 min, filaments started to emerge from the cell surface of the host, and fully secreted phages could be detected after 150 min (Supplementary Figure S1).

For the growth curve, the phage titre rose to roughly 1 × 10^9^ PFU/mL at 7 h post-infection and increased further overnight until it reached a plateau at approximately 2 × 10^10^ PFU/mL, which is in good agreement with the maximal titre that we regularly determined after phage propagation. As PMBT54 does not lyse its host, the production of virions is tightly linked to the growth of the host cells and likely stops when the host enters the stationary growth phase. Indeed, we found no active growth of the host after overnight incubation, indicating that *P. carnis* had entered the stationary phase by that time (Figure 2B). Interestingly, infection with PMBT54 had no effect on the growth dynamics of its host, as the CFU count rose identically between the infected culture and the uninfected control, suggesting that there is no significant growth defect associated with an infection under the tested conditions. Similar results have been observed for other filamentous phages and showed that host growth is only affected under certain conditions, like low salinity [30].

Filamentous Pf phages that infect *P. aeruginosa* have been shown to promote biofilm formation by interacting with extracellular polymers to form a highly ordered liquid crystalline matrix that increases adhesion while reducing desiccation and antibiotic susceptibility, thus increasing the virulence of the host [31,32,33]. Similarly, biofilm formation by spoilage-relevant *Pseudomonas* species in the dairy industry has been a major concern, as it allows the bacteria to persist in the production pipeline and resist decontamination procedures [34]. Hence, we sought to determine whether the phage PMBT54 has similar enhancing effects on biofilm formation by the milk spoilage bacteria *P. carnis* and *P. lactis*.

For this, we quantified biofilm formation in a Caso and 50% milk medium by infected and uninfected host bacteria (Figure 2C). In general, biofilm formation was increased in milk compared to Caso broth, and *P. lactis* showed elevated levels of biofilm formation in both media compared to *P. carnis*. When either of the host strains was infected with the phage PMBT54, we observed higher quantities of the biofilm matrix, indicating that, indeed, PMBT54 promotes the formation and/or stability of the extracellular biofilm matrix and, thus, potentially provides an advantage to its host in persisting under various environmental conditions.

No significant difference between infected and uninfected cells was detected for *P. lactis* L1-92 in milk, suggesting that the effect of an infection might be dependent on the growth medium. On the other hand, *P. lactis* usually exhibits high proteolytic activity, causing casein and other milk proteins to degrade and agglutinate [35]. It is possible that the biofilm measurement was influenced by the presence of too many aggregates that were not removed during the washing steps, and that these were predominantly stained instead of the biofilm matrix. In any case, for both strains, we were able to detect a positive effect on biofilm formation at least in one medium, emphasising the physiological growth influence of the phage PMBT54 on its host.

### 3.3. Genomic Characterisation

**Figure 3:**
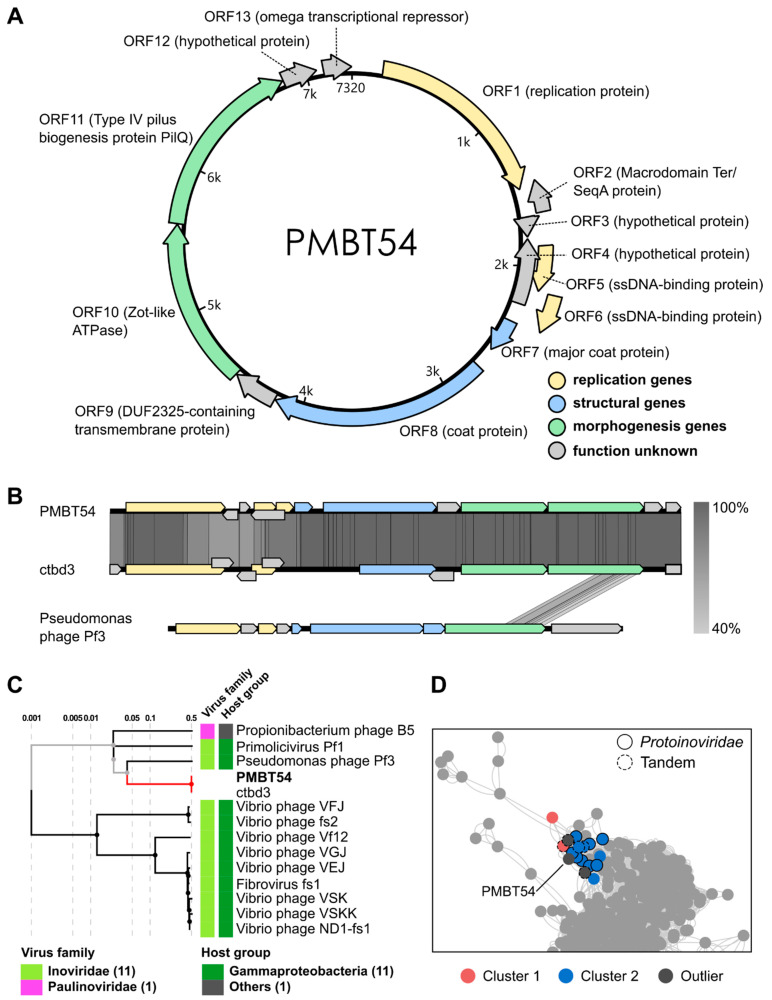
Genome map and taxonomic classification of PMBT54: (**A**) Circular genome of PMBT54, including annotated ORFs and predicted gene functions. Functional modules are highlighted in different colours. (**B**) Genome similarity between PMBT54 and the published genomes of phage ctbd3 (Accession: MH616883) and phage Pf3 (Accession: NC_001418). Comparison was performed with Easyfig. The sequence start positions of phages ctbd3 and Pf3 were set upstream of the replication module for easier comparison. Darker lines between genomes indicate a higher degree of similarity (see scale). (**C**) Proteomic tree of phages PMBT54 and ctbd3 using VipTree. Shown are all phages that displayed a similarity score above zero. (**D**) vContact2 cluster analysis using PMBT54 and inoviral sequences identified by Roux et al., 2019 [6]. Dots represent single genomes. Red and blue represent two clusters with which PMBT54 was associated as an outlier. PMBT54 was additionally associated with two other outlier genomes. Outlined circles were directly associated with PMBT54. Of those, solid lines refer to genomes classified in the *Protoinoviridae* family, and dashed lines represent tandem genomes.

The filamentous phage PMBT54 harbours a small circular genome of 7320 bp with a mol% GC content of 48.37. Using the RASTtk pipeline for annotation, 13 open-reading frames (ORF) could be identified, with two located on the (−) strand. Gene function was predicted using the HHsuite pipeline [19] (for alignment results, see Supplementary Table S3). Like most inoviruses, the genome of PMBT54 was organised into three functional modules containing replication, structural, and morphogenesis genes [4], respectively (Figure 3).

The genes of the replication module (indicated in yellow, Figure 3A,B) consist of ORF1 encoding a replication protein, which in most inoviruses is responsible for initiating replication [8], and two ORFs (5 and 6) encoding ssDNA-binding proteins. These ssDNA-binding proteins likely bind to newly synthesised (+) ssDNA molecules to prevent them from transitioning into the double-stranded replicative form, therefore rendering them ready for packaging into virions. Three additional ORFs, i.e., ORF2, -3, and -4 coding for hypothetical proteins of unknown function, are located within the replication module, of which two (ORF2 and ORF4) are antisense ORFs. ORF2 showed significant hits for both macrodomain Ter and SeqA proteins, both of which play different roles in bacterial replication [36,37]. Thus, although the exact function of ORF2 remains elusive, our results indicate an involvement of the gene in the replication of phage PMBT54, which is consistent with its location within the replication module.

The structural module (indicated in blue colour in Figure 3A,B) encompasses two ORFs encoding putative coat proteins: The product of ORF7 shows high similarity to the major coat protein of Xanthomonas phage Xf (PDB entry 2IFO; see Supplementary Table S3), which represents the main structural component and forms the multimeric filament that packages the phage’s genome [38]. We inferred a similar function of the product of ORF7 for the phage PMBT54. ORF8 was found to be similar to a putative inoviral coat protein that was identified in a metagenomic study [39], but its exact function remains cryptic.

ORF9 codes for a DUF2325-domain-containing protein, which, due to its predicted transmembrane binding properties, could be part of the structural module or, alternatively, be involved in morphogenesis of the virion. Within the morphogenesis module, we could identify two ORF products that are homologous to known proteins. Firstly, a zonula occludens toxin (Zot), which is widespread in inoviral genomes and is associated with phage assembly and secretion [8]. In *Vibrio* species, Zot is a secreted protein displaying gastrointestinal toxicity and is transferred by filamentous phages [40,41]. However, while the toxin itself carries the conserved Zot domain in its N-terminus, the Zot domain is not the toxic component: the C-terminal domain is cleaved from the Zot protein and conveys cytotoxicity against human epithelial cells [42]. The N-terminal domains of Zot-like phage ORFs are required for assembly and extrusion of the virion at the cell membrane. The presence of Walker A (GxxxxGK[S/T]—where x is any amino acid) and Walker B (hhhhDE—where h is any hydrophobic amino acid) motifs is typical for Zot homologs and represents the enzymatic basis for their ATPase activity, which is crucial during morphogenesis. These Walker motifs are present in most species of inoviruses found to date [43]. We identified full Walker A and Walker B motifs in the Zot homolog of PMBT54 (GKPGHGKS [position 8–15] and VAILDE [position 74–79], respectively), indicating that ORF10 codes for an active Zot-like ATPase involved in virion assembly. ORF11, on the other hand, was annotated as a type IV pilus protein belonging to the PilQ family. This protein is likely involved in secretion of the virion, as PilQ homologs have been shown to act like secretins in Ff phages, forming channels in the outer membrane of the host and, thus, enabling phage egress [44,45].

The functions of the hypothetical proteins encoded by ORF12 and ORF13 remain unknown. We could not find any significant database entry hit for ORF12, whereas ORF13 displayed high similarity to the omega transcriptional repressor found in various bacterial plasmids and was characterised by the presence of a ribbon–helix–helix DNA-binding motif. Plasmid-encoded homologs of the repressor are involved in plasmid maintenance and copy number control [46]. Considering that PMBT54 is likely sustained as an episome in the cell, ORF13 might convey a similar role.

For the taxonomic classification of PMBT54, we first checked whether PMBT54 showed any similarities to entries in the NCBI Blast database (https://blast.ncbi.nlm.nih.gov/Blast.cgi, (accessed on 8 March 2023)), and we found a closely related inovirus genome termed ctbd3 (coverage: 100%, identity: 97.07%, accession: MH616883, Figure 3B), which had been previously sequenced as a part of a metagenomic study from various animal samples but had not yet been isolated [39].

We constructed a proteomic tree using VipTree [20] (Figure 3C), which utilises a genome-wide tBlastx approach to identify closely related species in the VirusHost database [21]. Expectedly, a close similarity was observed to ctbd3, which we included in the analysis. A weak relatedness was also detected for Pseudomonas phage PF3 (S_G_ = 0.0458), which was mainly based on sequence similarities within PilQ (Figure 3B). Further species that matched with a lower tBlastx score (S_G_ < 0.02) belonged to the *Inoviridae* family, infecting mainly *Gammaproteobacteria*, and a *Propionibacterium* phage belonging to the *Paulinoviridae* family (Figure 3C).

To get a better understanding of the taxonomy of phage PMBT54, we used the vContact2 pipeline, which generates viral clusters based on protein similarities. We conducted the analysis on PMBT54 against the integrated ICTV and RefSeq Virus database. However, PMBT54 did not cluster with any phage species present in the databases. Therefore, we sought to extend the analysis to genome sequences published in a comprehensive study that mined a broad range of microbial hosts and shotgun metagenomes for inovirus-like sequences [6]. The study proposed a new classification of inoviruses into one viral order and six distinct families. To reveal the association of PMBT54 with the proposed families, we repeated the viral cluster analysis using vContact2 and the metagenomic phage sequences published by [6] (Figure 3D). PMBT54 was associated with two viral clusters, where genomes in one cluster are considered to be related on the genus level. However, PMBT54 only clustered as an outlier, which indicates a more distant relationship on the family or subfamily level.

The genomes in those clusters mainly belonged to the newly proposed family *Protoinoviridae*, which infects bacterial hosts of the *Gamma*- and *Betaproteobacteria* and, thus, are consistent with the host spectrum of PMBT54. Additionally, PMBT54 clustered with five genomes that could not be classified as one of the newly proposed inovirus families. Those unclassified genomes were labelled as “Tandem” [6]. Interestingly, the tandem genomes were exclusively identified in inoviral prophages of various *Pseudomonas* species, showing related host groups of PMBT54 and the tandem sequences.

## 4. Conclusions

In this study, we describe the novel filamentous phage PMBT54 infecting the milk spoilage bacteria *P. carnis* and *P. lactis*, and we show potential implications for biofilm formation by its host strains.

The phage was only distantly related to any described filamentous phages but showed a high similarity to the inoviral genome ctbd3, which was identified in a metagenomic study [39] but lacked physiological characterisation. PMBT54 displayed a modular genome organisation typical of the family *Inoviridae*, coding for replication, structural, and morphogenesis genes [9] (Figure 3A). Moreover, we assumed that the phage displays a non-integrative lifecycle but, rather, replicates as an episome in the host cell. While we did not unequivocally show this, the fact that the phage is constantly secreted from the host (Figure 2B), and that its circular genome could be extracted without prior induction methods used for prophages, points towards an episomal replication mechanism.

We were not able to detect a uniform virion size, but we hypothesise that a single infectious filament exhibits a size of approximately 1000 nm and that the phage is occasionally secreted as a multifilament virion consisting of conjoined filaments. In addition, we were not able to detect the presence of pili on the host strain *P. carnis* M132, indicating that the phage PMBT54 does not require pili for host cell attachment as described for many other *Inoviridae* [8]. Hence, more research is required for identifying the host receptor of phage PMBT54.

Finally, we provided evidence that infected host bacterial populations exhibit an improved capacity to form biofilms, which has potential implications for biofilm control in industrial settings. Strains that are infected with filamentous phages might generally form more stable biofilms and may thus persist in dairy plants. Tailored decontamination of those phages could therefore improve biofilm removal in critical areas. While we did not investigate the exact mechanism of how the phage PMBT54 improves the formation of biofilms, we presume a similar mechanism as described for filamentous phages infecting the pathogen *P. aeruginosa* [31,32,33], where the presence of the filaments in the extracellular biofilm matrix renders it more stable and resistant towards detrimental environmental influences.

## Data Availability

The complete genome sequence of phage PMBT54 is available at the NIH GenBank database (www.ncbi.nlm.nih.gov/genbank), under accession number OR047306.1.

## Supplementary Materials

The following supporting information can be downloaded at: https://www.mdpi.com/article/10.3390/v15091781/s1, Figure S1: Phage secretion from host *Pseudomonas carnis* M132: TEM images of the uninfected host and three timepoints post infection. Table S1: Pseudomonas strains used in this study [15,47,48,49,50,51]. Table S2: Top 3 Blast hits of assembled contigs. Node 3 was identified as a filamentous phage. Table S3: HMM alignments of the predicted ORFs against the databases UniProt, Pfam and PDB using HHsuite Table S4: Python script used to extract coding sequences from GenBank files and saving them as Fasta (.faa) files

## References

[1] Webster R.E. (1996). Biology of the filamentous bacteriophage. Phage Display of Peptides and Proteins.

[2] Rakonjac J., Bennett N.J., Spagnuolo J., Gagic D., Russel M. (2011). Filamentous bacteriophage: Biology, phage display and nanotechnology applications. Curr. Issues Mol. Biol..

[3] Frost L.S., Clewell D.B. (1993). Conjugative Pili and Pilus-Specific Phages. Bacterial Conjugation.

[4] Mai-Prochnow A., Hui J.G.K., Kjelleberg S., Rakonjac J., McDougald D., Rice S.A. (2015). ‘Big things in small packages: The genetics of filamentous phage and effects on fitness of their host’. FEMS Microbiol. Rev..

[5] Marvin D.A., Symmons M.F., Straus S.K. (2014). Structure and assembly of filamentous bacteriophages. Prog. Biophys. Mol. Biol..

[6] Roux S., Krupovic M., Daly R.A., Borges A.L., Nayfach S., Schulz F., Sharrar A., Matheus Carnevali P.B., Cheng J.-F., Ivanova N.N. (2019). Cryptic inoviruses revealed as pervasive in bacteria and archaea across Earth’s biomes. Nat. Microbiol..

[7] Loh B., Kuhn A., Leptihn S. (2019). The fascinating biology behind phage display: Filamentous phage assembly. Mol. Microbiol..

[8] Hay I.D., Lithgow T. (2019). Filamentous phages: Masters of a microbial sharing economy. EMBO Rep..

[9] Knezevic P., Adriaenssens E.M., Ictv R.C. (2021). ICTV Virus Taxonomy Profile: Inoviridae. J. Gen. Virol..

[10] Marvin D.A., Hohn B. (1969). Filamentous bacterial viruses. Bacteriol. Rev..

[11] Hill D.F., Short N.J., Perham R.N., Petersen G.B. (1991). DNA sequence of the filamentous bacteriophage Pf1. J. Mol. Biol..

[12] Takeya K., Amako K. (1966). A rod-shaped Pseudomonas phage. Virology.

[13] Knezevic P., Voet M., Lavigne R. (2015). Prevalence of Pf1-like (pro)phage genetic elements among *Pseudomonas aeruginosa* isolates. Virology.

[14] Lick S., Kröckel L., Wibberg D., Winkler A., Blom J., Bantleon A., Goesmann A., Kalinowski J. (2020). *Pseudomonas carnis* sp. nov., isolated from meat. Int. J. Syst. Evol. Microbiol..

[15] von Neubeck M., Huptas C., Glück C., Krewinkel M., Stoeckel M., Stressler T., Fischer L., Hinrichs J., Scherer S., Wenning M. (2017). *Pseudomonas lactis* sp. nov. and *Pseudomonas paralactis* sp. nov., isolated from bovine raw milk. Int. J. Syst. Evol. Microbiol..

[16] Volk V., Graw N., Stressler T., Fischer L. (2021). An indirect ELISA system for the detection of heat-stable *Pseudomonas* endopeptidases (AprX) in milk. J. Dairy Sci..

[17] Zhang Z., Schwartz S., Wagner L., Miller W. (2000). A greedy algorithm for aligning DNA sequences. J. Comput. Biol..

[18] Brettin T., Davis J.J., Disz T., Edwards R.A., Gerdes S., Olsen G.J., Olson R., Overbeek R., Parrello B., Pusch G.D. (2015). RASTtk: A modular and extensible implementation of the RAST algorithm for building custom annotation pipelines and annotating batches of genomes. Sci. Rep..

[19] Steinegger M., Meier M., Mirdita M., Vöhringer H., Haunsberger S.J., Söding J. (2019). HH-suite3 for fast remote homology detection and deep protein annotation. BMC Bioinform..

[20] Nishimura Y., Yoshida T., Kuronishi M., Uehara H., Ogata H., Goto S. (2017). ViPTree: The viral proteomic tree server. Bioinformatics.

[21] Mihara T., Nishimura Y., Shimizu Y., Nishiyama H., Yoshikawa G., Uehara H., Hingamp P., Goto S., Ogata H. (2016). Linking Virus Genomes with Host Taxonomy. Viruses.

[22] Sullivan M.J., Petty N.K., Beatson S.A. (2011). Easyfig: A genome comparison visualizer. Bioinformatics.

[23] Bin Jang H., Bolduc B., Zablocki O., Kuhn J.H., Roux S., Adriaenssens E.M., Brister J.R., Kropinski A.M., Krupovic M., Lavigne R. (2019). Taxonomic assignment of uncultivated prokaryotic virus genomes is enabled by gene-sharing networks. Nat. Biotechnol..

[24] Van Rossum G., Drake F.L. (1995). Python Reference Manual.

[25] Chapman B., Chang J. (2000). Biopython. SIGBIO Newsl..

[26] Shannon P., Markiel A., Ozier O., Baliga N.S., Wang J.T., Ramage D., Amin N., Schwikowski B., Ideker T. (2003). Cytoscape: A software environment for integrated models of biomolecular interaction networks. Genome Res..

[27] O’Toole G.A. (2011). Microtiter dish biofilm formation assay. J. Vis. Exp..

[28] Rakonjac J., Feng J.n., Model P. (1999). Filamentous phage are released from the bacterial membrane by a two-step mechanism involving a short C-terminal fragment of pIII. J. Mol. Biol..

[29] Cazares D., Cazares A., Figueroa W., Guarneros G., Edwards R.A., Vinuesa P. (2021). A Novel Group of Promiscuous Podophages Infecting Diverse Gammaproteobacteria from River Communities Exhibits Dynamic Intergenus Host Adaptation. mSystems.

[30] Goehlich H., Roth O., Wendling C.C. (2019). Filamentous phages reduce bacterial growth in low salinities. R. Soc. Open Sci..

[31] Secor P.R., Jennings L.K., Michaels L.A., Sweere J.M., Singh P.K., Parks W.C., Bollyky P.L. (2015). Biofilm assembly becomes crystal clear—Filamentous bacteriophage organize the *Pseudomonas aeruginosa* biofilm matrix into a liquid crystal. Microb. Cell.

[32] Secor P.R., Michaels L.A., Smigiel K.S., Rohani M.G., Jennings L.K., Hisert K.B., Arrigoni A., Braun K.R., Birkland T.P., Lai Y. (2017). Filamentous Bacteriophage Produced by *Pseudomonas aeruginosa* Alters the Inflammatory Response and Promotes Noninvasive Infection In Vivo. Infect. Immun..

[33] Secor P.R., Sweere J.M., Michaels L.A., Malkovskiy A.V., Lazzareschi D., Katznelson E., Rajadas J., Birnbaum M.E., Arrigoni A., Braun K.R. (2015). Filamentous Bacteriophage Promote Biofilm Assembly and Function. Cell Host Microbe.

[34] Quintieri L., Fanelli F., Caputo L. (2019). Antibiotic Resistant Pseudomonas Spp. Spoilers in Fresh Dairy Products: An Underestimated Risk and the Control Strategies. Foods.

[35] Maier C., Huptas C., von Neubeck M., Scherer S., Wenning M., Lücking G. (2020). Genetic Organization of the aprX-lipA2 Operon Affects the Proteolytic Potential of *Pseudomonas* Species in Milk. Front. Microbiol..

[36] Douraid D., Ahmed L. (2011). SeqA, the Escherichia coli origin sequestration protein, can regulate the replication of the pBR322 plasmid. Plasmid.

[37] Benza V.G., Bassetti B., Dorfman K.D., Scolari V.F., Bromek K., Cicuta P., Lagomarsino M.C. (2012). Physical descriptions of the bacterial nucleoid at large scales, and their biological implications. Rep. Prog. Phys..

[38] Marvin D.A. (1990). Model-building studies of Inovirus: Genetic variations on a geometric theme. Int. J. Biol. Macromol..

[39] Tisza M.J., Pastrana D.V., Welch N.L., Stewart B., Peretti A., Starrett G.J., Pang Y.-Y.S., Krishnamurthy S.R., Pesavento P.A., McDermott D.H. (2020). Discovery of several thousand highly diverse circular DNA viruses. Elife.

[40] Fasano A., Baudry B., Pumplin D.W., Wasserman S.S., Tall B.D., Ketley J.M., Kaper J.B. (1991). Vibrio cholerae produces a second enterotoxin, which affects intestinal tight junctions. Proc. Natl. Acad. Sci. USA.

[41] Pérez-Reytor D., Jaña V., Pavez L., Navarrete P., García K. (2018). Accessory Toxins of Vibrio Pathogens and Their Role in Epithelial Disruption during Infection. Front. Microbiol..

[42] Di Pierro M., Lu R., Uzzau S., Wang W., Margaretten K., Pazzani C., Maimone F., Fasano A. (2001). Zonula occludens toxin structure-function analysis. Identification of the fragment biologically active on tight junctions and of the zonulin receptor binding domain. J. Biol. Chem..

[43] Loh B., Haase M., Mueller L., Kuhn A., Leptihn S. (2017). The Transmembrane Morphogenesis Protein gp1 of Filamentous Phages Contains Walker A and Walker B Motifs Essential for Phage Assembly. Viruses.

[44] Conners R., McLaren M., Łapińska U., Sanders K., Stone M.R.L., Blaskovich M.A.T., Pagliara S., Daum B., Rakonjac J., Gold V.A.M. (2021). CryoEM structure of the outer membrane secretin channel pIV from the f1 filamentous bacteriophage. Nat. Commun..

[45] Conners R., León-Quezada R.I., McLaren M., Bennett N.J., Daum B., Rakonjac J., Gold V.A.M. (2023). Cryo-electron microscopy of the f1 filamentous phage reveals insights into viral infection and assembly. Nat. Commun..

[46] Murayama K., Orth P., La Hoz A.B.d., Alonso J.C., Saenger W. (2001). Crystal structure of omega transcriptional repressor encoded by *Streptococcus pyogenes* plasmid pSM19035 at 1.5 A resolution. J. Mol. Biol..

[47] Verhille S., Baïda N., Dabboussi F., Hamze M., Izard D., Leclerc H. (1999). *Pseudomonas gessardii* sp. nov. and *Pseudomonas migulae* sp. nov., two new species isolated from natural mineral waters. Int. J. Syst. Bacteriol..

[48] van den Beld M.J.C., Reinders E., Notermans D.W., Reubsaet F.A.G. (2016). Possible misidentification of species in the *Pseudomonas fluorescens* lineage as *Burkholderia pseudomallei* and *Francisella tularensis*, and emended descriptions of *Pseudomonas brenneri*, *Pseudomonas gessardii* and *Pseudomonas proteolytica*. Int. J. Syst. Evol. Microbiol..

[49] Reddy G.S.N., Matsumoto G.I., Schumann P., Stackebrandt E., Shivaji S. (2004). Psychrophilic pseudomonads from Antarctica: *Pseudomonas antarctica* sp. nov., Pseudomonas meridiana sp. nov. and Pseudomonas proteolytica sp. nov. Int. J. Syst. Evol. Microbiol..

[50] Hofmann K., Huptas C., Doll E.V., Scherer S., Wenning M. (2020). *Pseudomonas haemolytica* sp. nov., isolated from raw milk and skimmed milk concentrate. Int. J. Syst. Evol. Microbiol..

[51] Fiedler G., Gieschler S., Kabisch J., Grimmler C., Brinks E., Wagner N., Hetzer B., Franz C.M.A.P., Böhnlein C. (2022). *Pseudomonas rustica* sp. nov., isolated from bulk tank raw milk at a German dairy farm. Int. J. Syst. Evol. Microbiol..

