# Supplementary material for: Characterisation of the Novel Filamentous Phage PMBT54 Infecting the Milk Spoilage Bacteria Pseudomonas carnis and Pseudomonas lactis
Source: Viruses. 2023 Aug 22;15(9):1781. doi: 10.3390/v15091781 (PMC10534721; doi:10.3390/v15091781)
Supplement: Supplementary file 1 [file viruses-15-01781-s001.zip › viruses-2480829-supplementary.pdf]

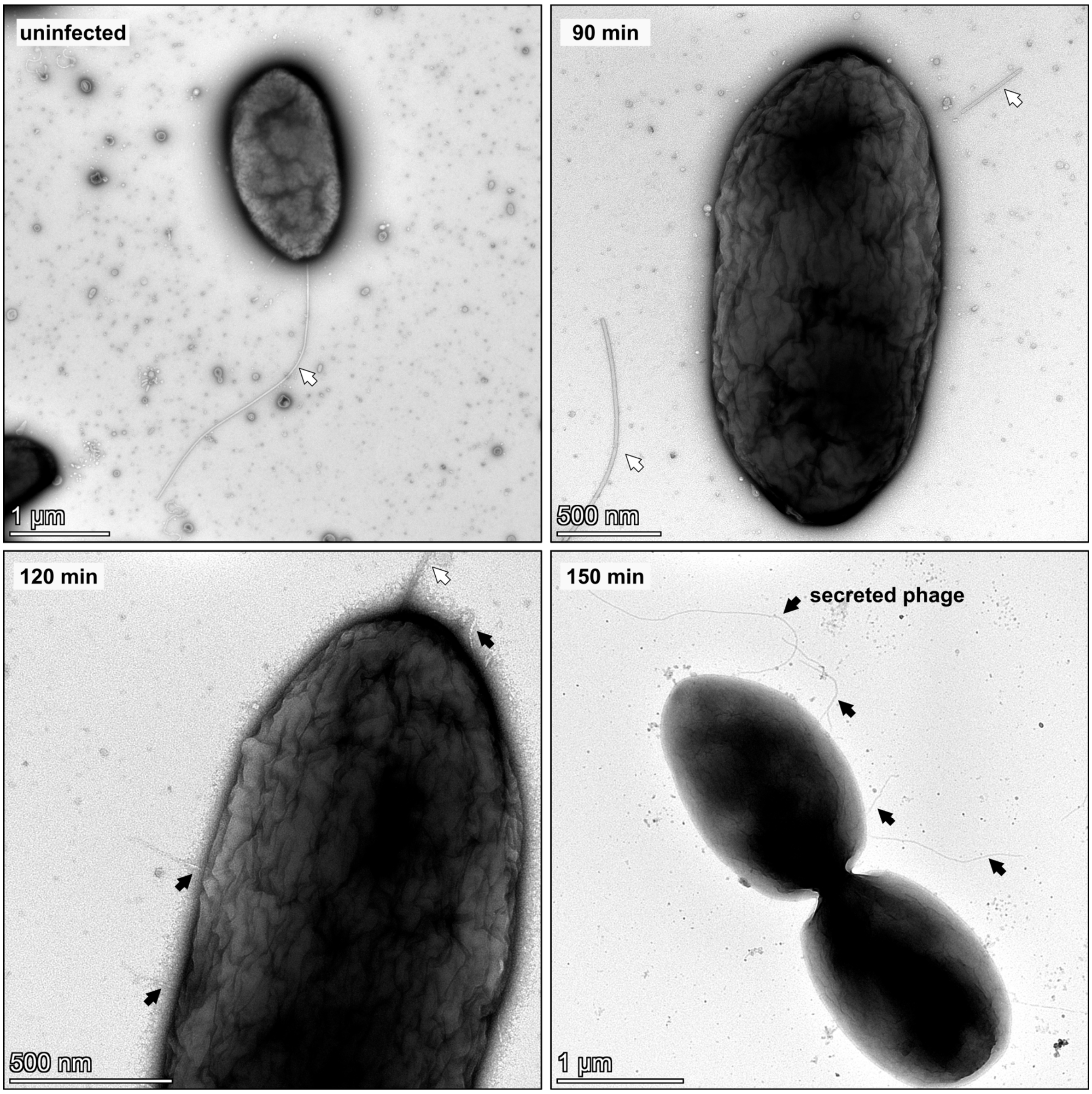

Figure S1. Phage secretion from host *Pseudomonas carnis* M132: TEM images of the uninfected host and three timepoints post infection. Bacterial flagella are indicated with white arrows. Black arrows indicate the filamentous phage.

Table S1. *Pseudomonas* strains used in this study.

Strain description	Origin	Source/ Reference[1,2]
P. fluorescens L1-82	raw milk	TUM isolate
P. fluorescens L1-83	raw milk	TUM isolate
P. fragi L1-85	raw milk	TUM isolate
P. gessardii L1-88 (DSM 17152)	mineral water	Verhille et al. (1999) [47] emend. van den Beld et al.(2016) [48]
P. gessardii L1-89	raw milk	TUM isolate
P. gessardii L1-90	milk semifinished product	TUM isolate
P. lactis L1-91	raw milk	TUM isolate
P. lactis L1-92	raw milk	TUM isolate
P. lactis L1-93 (DSM 29167)	raw milk	Neubeck et al. (2017) [15]
P. lundesis L1-95	raw milk	TUM isolate
P. lundesis L1-96	milk semifinished product	TUM isolate
P. meridiana L1-98	raw milk	TUM isolate
P. meridiana L1-99	raw milk	TUM isolate
P. protegens L1-101	raw milk	TUM isolate
P. protegens L1-102	raw milk	TUM isolate
P. proteolytica L1-103 (DSM 15321)	cyanobacterial mat samples	Reddy et al. (2004) [49] emend. van den Beld et al. (2016) [48]
P. proteolytica L1-104	raw milk	TUM isolate
P. proteolytica L1-105	raw milk	TUM isolate
P. proteolytica L1-106	raw milk	TUM isolate
P. proteolytica L1-107	cream	TUM isolate
P. haemolytica L1-177 (DSM 108987)	raw milk	Hofmann et al. (2020) [50]
P. iridis L1-222 (M19)	raw milk	MRI isolate
P. lundesis M47	raw milk	MRI isolate
P. rustica L1-223 (M59) (DSM 112348)	raw milk	Fiedler et al., 2022 [51]
P. sp. M70	raw milk	MRI isolate
P. bubulae M72	raw milk	MRI isolate
P. carnis M132	raw milk	MRI isolate

[1]TUM: Technical University of Munich. Strains have been isolated within the Industrial Collective Research (IGF) funded project AiF-FV 16588 N.

[2]MRI: Max Rubner-Institut. Strains have been isolated within the Industrial Collective Research (IGF) funded project AiF-FV 20027 N.

Table S2. Top 3 Blast hits of assembled contigs. Node 3 was identified as a filamentous phage.

Description	Scientific Name	Common Name	Taxid	Max Score	Total Score	Query cover	E Value	Per. Ident	Acc. Len	Accession
**NODE_1**										
Pseudomonas monteilii STW0522-72 plasmid pSTW0522-72-3 DNA,...	Pseudomona s	NA	76759	45142	69238	75%	0	99	44219	AP022476.1
Pseudomonas putida strain IEC33019 plasmid pIEC33019, complete...	Pseudomona s	NA	303	42125	76004	82%	0	99,3	52710	CP016446.1
Pseudomonas mendocina strain AOUC-01/15 plasmid pMEN15, comple.	Pseudomona s	NA	300	36221	71110	75%	0	100	55387	MK671727.1
**NODE_2**										
Pseudomonas fragi strain NMC25 plasmid unnamed2, complete…	Pseudomona s .	NA	296	13682	36520	68%	0	87,9	54359	CP021134.1
Pseudomonas migulae strain R1-9 plasmid unnamed, complete...	Pseudomona s	NA	78543	9768	35396	68%	0	87,3	68338	CP043572.1
Pseudomonas mandelii JR-1 plasmid, complete sequence	Pseudomona s .	NA	1147786	6482	7250	9%	0	100	410512	CP005961.1
**NODE_3 (=PMBT54)**										
Inoviridae sp. isolate ctbd3, complete genome	Inoviridae sp.	NA	2219103	11537	12845	100%	0	97,9	7321	MH616883.1
Pseudomonas tolaasii strain 2192T chromosome	Pseudomona s .	NA	29442	65,8	65,8	4%	0,0001	70,7	6856683	CP020369.1
Pseudomonas aeruginosa strain PAAK095 chromosome, complete genome	Pseudomona s	NA	287	60,2	120	1%	0,006	79,6	6780782	CP054473.1
**NODE_7**										
Uncultured prokaryote from Rat gut metagenome metamobilome,...	uncultured p..	NA	198431	1657	2711	55%	0	93,8	4028	LN853216.1
Pseudomonas aeruginosa strain YTSY4 plasmid pYTSY4-VIM, comple...	Pseudomona s .	NA	287	1530	2738	59%	0	91,2	14870	MT313930.1
Uncultured prokaryote from Rat gut metagenome metamobilome,...	uncultured p..	NA	198431	1391	1391	35%	0	88	2394	LN854271.1

Table S3. HMM alignments of the predicted ORFs against the databases UniProt, Pfam and PDB using HHsuite. Best hit and corresponding database is indicated next to the ORF number. Below are alignment statistics for each hit.

```
ORF1, DB = UniProt
>UniRef100_A0A0H3ZQY0 Replication protein, phage-associated n=1 Tax=Vibrio tasmaniensis TaxID=212663
RepID=A0A0H3ZQY0_9VIBR
Probab=100.00  E-value=1.4e-85  Score=664.82  Aligned_cols=372  Identities=42%  Similarity=0.745  Sum_probs=341.5
Template_Neff=3.155

Q ORF1              19 EAALGSIAKSSPTSDLK------VAKAARAQRYQALSQSRGWLKPLARKNPNKAYGWDLHRTQDCRYKRVGIGVTVHFDQ   92 (423)
Q Consensus         19 ~~~Lg~~~ks~~~~~~~~~------~~~~~r~~r~~l~~~a~~~~~~~~~~~~~~~~~~~~~~Rv~~Cg~~~~~~~v~v~~~~   92 (423)
                       .+|||+++||++|+.++      ..+..|.+||.|+...+..+.. .++++++.+....+||+.|.+..+...|.|..+.
T Consensus         36 g~pLg~~aksssp~~s~~~~~~~~r~~~~r~~R~~l~~~~r~~~~~~~-~~~~~~~~~~~~~~~~Rv~~C~~~~v~~~~~v~~~~  114 (514)
T UniRef100_A0A0   36 GEPLGITAKSSSPPPSTPAKRSERSRALRAERYALQILGRDVFKA-DPDNKNLQYWSNVHRVCKCHWVRIAPNVGVMRSV  114 (514)
Confidence               3599999999999733      4556778888888888855433 2345566778888999999999998778888777


Q ORF1              93 KHQTAFYGDVATCGSVWACPVCATIIQNRRRPEVQQLVDWAYDQGYQVTMLTFTFPHKAFDRLKFMLNSQADAFTRLRKG  172 (423)
Q Consensus         93 ~~~~a~~~gl~~Cgsvw~CPvCa~ris~~R~~el~~~~~~~~~~~~~~g~~~~~~lTLT~~H~~~d~L~~ll~~l~~A~~rl~~~  172 (423)
                       ++++|+|.|+.+|||||.||||++||+++|++||.++|.+++++|+.++|||||+||...+.|+|+|..|.+||++|++|
T Consensus        115 ~~~~a~y~gl~~CGSVW~CPvCsakI~erRr~Eia~~~~~~ay~~g~~~iMVTlT~pH~~~q~l~dll~~~~~A~~rlrsg  194 (514)
T UniRef100_A0A0  115 EHGRAHYSGLATCGSVWACPVCSAKIQERRRQEIAQAMDWAYEQGLQVIMVTLTAPHYSRQSLADLLEAQSKALKRLRSG  194 (514)
Confidence               7889999999999999999999999999999999999999999999999999999999999999999999999999


Q ORF1             173 NSWDLFRKRFGFVGLVRSLENTHGANGWHPHTHEIWITKDLTPVARGDFEAFVKRRWKMACIKAGLLDDTNKAQVFAFDL  252 (423)
Q Consensus        173 r~~k~~~~~~gi~G~iRa~EVT~g~nGWHPH~HvLl~~~~~~~~~~~~~~~~~~~~~~~~~l~~~W~~al~~~Gl~~~~~~~~~~~~~~~~~~~~~~~  252 (423)
                       +.|+.++.++|+.|+||++|||+|.||||||+|.|+|++.+.+.+  .+..+..++|.++|.++||..-.   ++.+|+.
T Consensus        195 k~w~~lk~~~g~~G~iralEvT~G~NGWH~HtHelw~v~~~ad~~---~lk~~~~~rW~~ac~~~Gl~~~~~~---------fr~  269 (514)
T UniRef100_A0A0  195 KYWRSLKGRYGFQGLIRALEVTHGQNGWHPHTHELWFVRGDADAE--ALKEEVLRRWEAACQRAGLLPAG---KVRAFRE  269 (514)
Confidence               9999999999999999999999999999999999999988755  7779999999999999987432   6789999


Q ORF1             253 HAVDVRYEVSTSDYLVKQDNSRKWGVDREMTAQAAKVAPDGVTPTVHPHEFLI-RQS---EGDDARYREYVTGMHGKRQL  328 (423)
Q Consensus        253 hgvdvr~~~~~a~~YlaK~~~~~~~~wg~~~E~tk~~~K~gr~~~~~~g~TPf~lL~-~~~~~~~~~~~~~~l~~Ey~~a~~GrR~l  328 (423)
                       |.|||+....+++|++|+|++..||+..|||+++.|.||+   +|+|||+||. +..   ..+.++|.||+.+|||+|||
T Consensus        270 ~avd~k~~~~~~seYlaK~dd~~hWGadrE~a~~~aK~gr~---kG~hPf~LLa~~~~~~g~~~~~~~~~~~~eya~A~KGrrQ~  346 (514)
T UniRef100_A0A0  270 HAVDIKDNASCSEYLAKQDDSKHWGADREIAKGSAKRGRK---KGFHPFQLLAEDEGDGDDDAAERFTEYAKAMKGRRQL  346 (514)
Confidence               9999998889999999999999999999999999998   8999999999 332   5677999999999999999


Q ORF1             329 YWSQGLKDLCGLRDVSDEEIAKEHTEKADLLAHLTDAQWNVVRGNDALSELLDAAERGGWPAICSLLNSLG  399 (423)
Q Consensus        329 ~wS~gLk~~l~l~e~~Deeia~e~~~~~~~~~~v~~i~~~~~W~~i~~~~~~~~~~~lL~~aE~~g~~~v~~~l~~~~  399 (423)
                       +||+|||+.++++|.+||||++++.++.+.|..|+.+.|.+.+.+++||+++|.+|+.++.+||..+.
T Consensus        347 ~WS~GLK~~~gi~E~sDEeia~e~~~~~~E~l~~i~~~~~W~~i~~~~~~ra~lLd~aE~~g~a~~~~~l~d~~  417 (514)
T UniRef100_A0A0  347 FWSPGLKDRAGIEEKSDEEIAEEEADEMEELLSIEHEDWRRISKLGARAELLDAAETGGFAGAAAWLRDWW  417 (514)
Confidence               99999999999999999999999877899999999999999999999999999999999999999986
```

```
ORF2, DB = PDB
>3VEA_A Macrodomain Ter protein/DNA complex; macrodomains, chromosome, DNA condensation, DNA; 2.55A {Yersinia pestis}
Probab=92.51  E-value=0.045  Score=37.40  Aligned_cols=37  Identities=24%  Similarity=0.424  Sum_probs=33.7
Template_Neff=6.100

Q ORF2              5 RLKNISVSKEVWTQLKLLEAEKDCTHTQVLEYLLQRS   41 (64)
Q Consensus         5 rlknisvskevwtqlklleaekdcthtqvleyllqrs   41 (64)
                      +.++|+++.++|..|+-+-...+||.++++++++...
T Consensus        90 ~~ksitLt~~A~~~Ls~lA~~~g~TlSe~Ie~lL~~~  126 (151)
T 3VEA_A           90 RKKSIDLEFLVWQRLAVLARRRGNTLSDTVVQLIEDA  126 (151)
T ss_dssp              CEEEEEEEHHHHHHHHHHHHHHHTCCHHHHHHHHHHHH
T ss_pred              CCceeeCcHHHHHHHHHHHHHhCCCHHHHHHHHHHHH
Confidence            4678999999999999999999999999998653
```

```
ORF3, DB = PDB, Pfam, UniProt
no significant results
```

```
ORF4, DB = PDB, Pfam, UniProt
no significant results
```

```
ORF5, DB = PDB
>1PFS_B PF3 SINGLE-STRANDED DNA BINDING PROTEIN; DNA-BINDING PROTEIN, VIRAL, BACTERIOPHAGE PF3; NMR {Pseudomonas phage
Pf3} SCOP: b.40.4.7
Probab=98.29  E-value=1.8e-09  Score=69.05  Aligned_cols=75  Identities=27%  Similarity=0.515  Sum_probs=71.2
Template_Neff=6.500

Q ORF5              7 ATIELTGQNKTGTSMKSGAPKPWWRLGAYVQVPGLLHPQAIEFFTSEVTQVQPAGKYEAPCATRIQDGKILVEPDLSQLK   86 (94)
Q Consensus         7 atieltgqnktgtsmksgapkpwwrlgayvqvpgllhpqaiefftsevtqvqpagkyeapcatriqdgkilvepdlsqlk   86 (94)
                      .+|||++++|+|++ |+|  +||+||+||+|++|.+||++|+|+|+   ++||||.|+|++++.|++||||++++|+++|+
T Consensus         3 i~IeI~~~~r~g~s-k~G--kpy~i~~ay~~l~g~~yP~~~~i~l------y~~G~Y~l~~~~~v~~Grl~~~~~~~~~~~~   76 (78)
T 1PFS_B            3 IQITFTDSVRQGTS-AKG--NPYTFQEGFLHLEDKPHPLQCQFFVE---SVIPAGSYQVPYRINVNNGRPELAFDFKAMK   76 (78)
T ss_dssp              EEEEEEEEEEEEEC-TTS--CEEEEEEEEECCTTCSSCEEEEEECS---SCCCSEEEEEEEEEEETTEEEEEECTTSCE
T ss_pred              eEEEEecceEEeec-CCC--CeeEEEEEEEEEeCCCCCcEEEEEECC---CCCCCEEEEEcCEEEEeCCCeEEEechhHhc
Confidence            57999999999999 899  99999999999999999999998   8999999999999999999999999986


Q ORF5             87 A   87 (94)
Q Consensus        87 a   87 (94)
                      .
T Consensus        77 ~   77 (78)
T 1PFS_B           77 R   77 (78)
T ss_dssp              E
T ss_pred              c
Confidence            4
```

```
ORF6, DB = Pfam
>PF17091.8 ; Inovirus_G7P_1 ; Inovirus G7P protein
Probab=97.16  E-value=8.8e-07  Score=47.29  Aligned_cols=36  Identities=11%  Similarity=0.040  Sum_probs=34.4
Template_Neff=5.600

Q ORF6             38 LDPAQLGVAFGAGFTLVTAFLVFGLGVRVLLNFIKT   73 (74)
Q Consensus        38 ldpaqlgvafgagftlvtaflvfglgvrvllnfikt   73 (74)
                      .|+++++++|++||++|++||++|+++|+|++|||+
T Consensus         2 pd~~~~a~~f~~gF~lv~~~ylva~~~G~Vl~~~~~~   37 (38)
T COAT3_XANCP/1-    2 PSQEDAVAWSTGCCGLVIVWFVLGRLAGSVAGMFND   37 (38)
T ss_pred              CCHHHHHHHHHHHHHHHHHHHHHHHHHHHHHHHHhhcC
Confidence            378999999999999999999999999999996
```

```
ORF7, DB = Pfam
>PF05356.14 ; Phage_Coat_B ; Inovirus Coat protein B
Probab=81.06  E-value=0.24  Score=27.27  Aligned_cols=44  Identities=25%  Similarity=0.238  Sum_probs=33.2
Template_Neff=5.800

Q ORF7             29 AAKWDYTQITTGVDFSGIATGVLSVAAVLAGIYAGIKGARIVLGFL   74 (76)
Q Consensus        29 aakwdytqittgvdfsgiatgvlsvaavlagiyagikgarivlgfl   74 (76)
                      +.--|-+.+++.++  +..+.+.++.....+++..+++.|.+.+++
T Consensus        13 a~a~d~t~v~t~I~--------i~~iG~avl~v~v~i~~~k~vrr~~   56 (56)
T I3CK69_9GAMM/1   13 VANITVTDIVDQLK--AGAPAIVSIATAAISLLAVIAVFKYVRGAL   56 (56)
T ss_pred              hhccCHHHHHHHHH--hhHHHHHHHHHHHHHHHHHHHHHHHHHHHHhhC
Confidence            34456777877665  677778888888889999998876653
```

```
ORF8, DB = UniProt
>UniRef100_A0A345MS05 Putative coat protein A n=1 Tax=Inoviridae sp. TaxID=2219103 RepID=A0A345MS05_9VIRU
Probab=100.00  E-value=5.2e-53  Score=405.60  Aligned_cols=327  Identities=99%  Similarity=1.548  Sum_probs=306.2
Template_Neff=1.000

Q ORF8            157 GTEGSVIDSKGKKYVLSRASPDSICTSSCQYKEPRATTCYLLEGSKTRGFCNYIYQLQKDPEDTKYPQVCNTYTDYDDPE  236 (483)
Q Consensus       157 ~~~~~v~~~~G~~~v~~~~~~p~svC~ngC~y~~~~~~~~~c~~~~gs~~~gfCny~~~~~~~~~~~~~~~~~tG~~C~~~~~~~~~~~g~  236 (483)
                      .+...|+.+.+|.||+++++|.++|..+|||+.++.+.||++.++++++||||+|+|+++++.+|..+.|++.+.+..++
T Consensus         2 gtegsvidskgkkyvlsraspdsictsscqykeprattcyllegsktrgfcnyiyqlqkdpedtkypqvcntytdyddpe   81 (328)
T UniRef100_A0A3    2 GTEGSVIDSKGKKYVLSRASPDSICTSSCQYKEPRATTCYLLEGSKTRGFCNYIYQLQKDPEDTKYPQVCNTYTDYDDPE   81 (328)
Confidence            34567788899999999888999999999999999999999999999999999999999999999999999997799999


Q ORF8            237 QGDPLNPSDETGGTDDPGDETPTDPEDGGSGNSGCTGTNCEGNNQGGGDESGNTGGGSGSGGSEGGSTGGGTGSGGSDGG  316 (483)
Q Consensus       237 ~g~~~~p~~~~~~~p~dp~d~Cp~Gy~~~wsG~ttC~~~~~~dp~dp~~p~~~~~~~~~~~g~~~~~~~~~~~~g~~~~~~~~~~~~~~~~g~  316 (483)
                      .++|+||.++....+||.|+-|...+|.-+++..|..++++..+.....-+..++++|++.++..||.+++|+|+++..|+
T Consensus        82 qgdplnpsdetggtddpgdetptdpedggsgnsgctgtncdgnnqgggyesgntgggsgsggseggstgggtgsggsdgg  161 (328)
T UniRef100_A0A3   82 QGDPLNPSDETGGTDDPGDETPTDPEDGGSGNSGCTGTNCDGNNQGGGYESGNTGGGSGSGGSEGGSTGGGTGSGGSDGG  161 (328)
Confidence            99999999999999999999999999999998899999999999876665444488899999999999999999999999999


Q ORF8            317 GSTGGTGDGETPGTGTGGGQGGGTGTGGGSGNGSGSGGSPGTGSGSGSSGGGSSGSGEGDGSGSGDGLGSFESPGDLTLS  396 (483)
Q Consensus       317 ~~~gg~g~~~~~~g~~~~~~g~g~~~~~~~~~~~~~~~~~~~~~~~~~~~g~~g~g~g~g~g~g~~~~~~~~~~g~~~~g~~~~~~~~~~~f~~~~~~~~~~~~  396 (483)
                      ++.||+|++...|.++++|.|++.++++++++|++.+|+.|.|.|+|+|+.+|++++.|||++.|.-|+.-+|.+|+.|.|.
T Consensus       162 gstggtgdgetpgtgtgggqgggtgtgggsgngsgsggspgtgsgsgssgggssgsgegdgsgsgdglgsfespgdltls  241 (328)
T UniRef100_A0A3  162 GSTGGTGDGETPGTGTGGGQGGGTGTGGGSGNGSGSGGSPGTGSGSGSSGGGSSGSGEGDGSGSGDGLGSFESPGDLTLS  241 (328)
```

```
Confidence           99999999999999999999999999999999999999999999999888888888888888888888888889999999999


Q ORF8           397 TDQLQQRSESAATRYAASYKQTQTFQAINQFSGESHSGTCPTGSVELFDQTIIFDGHCKLFDTVKPTLEVVFKAAWSILG  476 (483)
Q Consensus      397 ~~~~~~~~~~~~~~~~~~~~~spl~~~l~~f~~~s~~GsCP~~t~~~fg~~~tfD~HC~l~e~ir~~l~avfla~wallA  476 (483)
                     .+.|.++.++....+.+..|+...+++|..|+.-+|++.||..++.||.++|+||-||.||+.+++.|..||-|+|.||.
T Consensus      242 tdqlqqrsesaatsyaasykqtqtfqainqfsgeshsgtcptgsvelfdqtitfdghcklfdtvkptlevvfkaawsilg  321 (328)
T UniRef100_A0A3  242 TDQLQQRSESAATSYAASYKQTQTFQAINQFSGESHSGTCPTGSVELFDQTITFDGHCKLFDTVKPTLEVVFKAAWSILG  321 (328)
Confidence           99999999998888899999999999999999999999999999999999999999999999999999999999999999


Q ORF8           477 VIIILTA  483 (483)
Q Consensus      477 ~rIvLsA  483 (483)
                     |.|||.|
T Consensus      322 viiilta  328 (328)
T UniRef100_A0A3  322 VIIILTA  328 (328)
Confidence           9999986
```

```
ORF9, DB = Pfam
>PF10734.12 ; DUF2523 ; Protein of unknown function (DUF2523)
Probab=95.55  E-value=0.00026  Score=40.83  Aligned_cols=54  Identities=22%  Similarity=0.359  Sum_probs=50.8
Template_Neff=8.000

Q ORF9            34 LWTGFLQMLDSLGIAEAVRNASSMFSFIPETVWYFANMAQVQYGIGVVLGALAIRFLIR   92 (98)
Q Consensus       34 lwtgflqmldslgiaeavrnassmfsfipetvwyfanmaqvqygigvvlgalairflir   92 (98)
                     -.++++.++|++     .++++++++++|+++.++++++|+|+|+++++||+++|+++|
T Consensus       26 t~~~~~~~i~~~~~~~~~~~~i~~~~~~~lp~~~~~~~l~~~gi~~~l~ii~sA~~~r~~l~   79 (79)
T H2FW36_OCESG/7  26 VYQGADFMLSQL-----ISKAQSLWNGLPSHIIQILAMAGVNEAISIGLSGVVFHVTMS   79 (79)
T ss_pred           HHHHHHHHHHHH-----HHHHHHHHcCCCHHHHHHHHHcCcHHHHHHHHHHHHHHHHHhC
Confidence           346888999999     8999999999999999999999999999999999999986
```

```
ORF10, DB = Pfam
>PF05707.15 ; Zot ; Zonular occludens toxin (Zot)
Probab=97.39  E-value=2.4e-07  Score=65.98  Aligned_cols=179  Identities=21%  Similarity=0.319  Sum_probs=130.2
Template_Neff=11.300

Q ORF10            3 IHAYVGKPGHGKSYGVVERVVIPSLKQNRHVVTNIP-LNADDLLADFGGNITQ------L----PEDWFDLEDMAELFPP   71 (368)
Q Consensus        3 ihayvgkpghgksygvvervvipslkqnrhvvtnip-lnaddlladfggnitq------l----pedwfdledmaelfpp   71 (368)
                     +++++|++|+|||.++..+++....+.+.+++++.. .....+...+. .+..       .     ......+..+.+...+
T Consensus        2 ~~li~G~~GsGKt~~~~~~~~~~~~~~~~~~~~~~~~~~~~~~~~~~~~~~~~-~~~~~~~~~~~~~~~~~~~~~~~~~~~~~~~   80 (210)
T ZOT_VIBCH/2-21   2 IFIHHGAPGSYKTSGALWLRLLPAIKSGRHIITNVRGLNLERMAKYLK-MDVSDISIEFIDTDHPDGRLTMARFWHWARK   80 (210)
T ss_pred           eEEEEcCCCCChhHHHHHHhHHHHHHHcCCEEEEcCCCCCHHHHHHHhc-CCCCceEEEEEcCCCcccchhhHHHHHcc
Confidence           6889999999999999876233344445666655 44444433322 1111      0    1223334444444455


Q ORF10           72 GCVAILDEVWRRWPRGQRSNMA-------------NKLDQSLLAEHRHKVDARNNSMRIVLVTQDLQQLANWACLLIET  137 (368)
Q Consensus       72 gcvaildevwrrwprgqrsnma-------------nkldqsllaehrhkvdarnnsmrivlvtqdlqqlanwaclliet  137 (368)
                     +.++++||++.+++........               ...+..++...++      .+..++++++.++.+++.++++++.
T Consensus       81 ~~~iiiDe~~~l~~~~~~~~~~~~~~~~~~~~~~~~~~~~~~~~~~~~~~~-------~~~~vi~~~~~~~~~~i~~~~~~~~~~  154 (210)
T ZOT_VIBCH/2-21  81 DAFLFIDECGRIWPPRLTVTNLKALDTPPDLVAEDRPESFEVAFDMHRH------HGWDICLTTPNIAKVHNMIREAAEI  154 (210)
T ss_pred           CCEEEEeCchhccCcccccCChhhcCCCCccccccCCChhHHHHHHHHhc------cceEEEEEcCCHHHHHHHHHHHHHh
Confidence           699999999998877643222             3345566667777      788999999999999999999999


Q ORF10          138 FRVVKLS----KKVYRVDVYNGSVTGDKPSQKNLIRQYPGKFSSDIHRYYSSATQS  190 (368)
Q Consensus      138 frvvkls----kkvyrvdvyngsvtgdkpsqknlirqypgkfssdihryyssatqs  190 (368)
                     ++.+.+++    .++|..+.|.+++++.+++  ++.+.+++++++++|++|+|+++.
T Consensus      155 ~i~~~~~~~~~~~~~~~~~~~~~~~~~~~~~~~~~~~~~~~~~~-~~~~~~~~~~~~~~~~~~~~~~~y~s~~~~~~  209 (210)
T ZOT_VIBCH/2-21 155 GYRHFNRATVGLGAKFTLTTHDAANSGQMDS--HALTRQVKKIPSPIFKMYASTTTG  209 (210)
T ss_pred           eEEEEehhhcCCCCeEEEEEeccccCCCCCc--ccccccceeCChHHhhcceeccCC
Confidence           99999998    5678888889998888777  78899999999999999999874
```

```
ORF11, DB = PDB
>6W6M_K Type IV pilus secretin PilQ; secretion system, outer membrane protein;{Vibrio cholerae}
Probab=100.00  E-value=5.6e-43  Score=316.21  Aligned_cols=382  Identities=29%  Similarity=0.484  Sum_probs=315.7
Template_Neff=9.500

Q ORF11           22 ADEPLTFDFGSIQTSDALQLIADYANKNIVVADDVSGAISLRMKDVTWEQALDYVVLRKQLHQFESDGVIFVSRNKDYFS  101 (409)
Q Consensus       22 adepltfdfgsiqtsdalqliadyanknivvaddvsgaislrmkdvtweqaldyvvlrkqlhqfesdgvifvsrnkdyfs  101 (409)
                     .+++++++|++++|+++|+.|++.+|+||++++++.+++++++++++|++||++|+..+||.|.+++++++|.+..+...
T Consensus      155 ~~~~isl~~~~~~l~~vl~~la~~~g~nivi~~~v~g~vt~~l~~~~~~~~~~aL~~il~~~gl~~~~~~~~~~i~i~~~~~~~~~~~  234 (571)
T 6W6M_K         155 EGKLISINFQDIPVRNVLQLIADYNGFNLVVSDSVVGNLTLRLDGVPWQQVLDIILQVKGLDKRVDGNVILIAPKEELDL  234 (571)
T ss_dssp            -----CEEEEEEEHHHHHHHHHHTTSCCEECCSSCSCEEEEEEECSSCHHHHHHHHHHHTCCEEEECSSSEEEECHHHHHH
T ss_pred            CCceEEEEeecCCHHHHHHHHHHHCCEEEECCCCCCeEEEEEcCeeHHHHHHHHHHHcCCCcEEEcCCEEEEeehhHHhH
Confidence           45789999999999999999999999999999999999999999999999999999999999999999999998877554


Q ORF11          102 GDETAQG-YGSGGSHSPKKKVVYPMSLHKVRYITAAEAIKGFPLA---QGDDGVETLQSNEQANVIVARLAPERVAELRQ  177 (409)
Q Consensus      102 gdetaqg-ygsggshspkkkvvypmslhkvryitaaeaikgfpla---qgddgvetlqsneqanvivarlapervaelrq  177 (409)
                     ....... ........+..+      ..++++|.+++++.++..    ..+++.+.+.+|+++|+|+|+++|+|+.+++|++
T Consensus      235 ~~~~~~~~~~~~~~~~~~~~~~-----~~~~~~~~l~~~~~~~~~~~~~~~~~~~~l~~~~g~i~~d~~tn~liV~~~~~~~~~~~i~~  309 (571)
T 6W6M_K         235 REKQALEKARLAEELGDLKS-----EIIKINFAKASDIAAMIGGEGNVNMLSERGSISIDERTNSLLIRELPDNIAVIRE  309 (571)
T ss_dssp            HHHHHHHHHHHHHHSCCCEE-----EEEECCSSCHHHHHHHHSCSSTTTSSCSSCEEEEETTTTEEEEEECTTHHHHHHH
T ss_pred            HHHHHHHHHHHHHHhcccee-----EEEEeccccCHHHHHHHhcCCCceecccCCceEEEecCCCEEEEEeCHHHHHHHHH
Confidence           3322222 122223345567     889999999999998721   1678889999999999999999999999999


Q ORF11          178 YLSMIDVIRRQVMIEARIVEVDTSYTKSLGVNWRGSIGIGDVT--AAVNS---------------------QLSAISNAV  234 (409)
Q Consensus      178 ylsmidvirrqvmiearivevdtsytkslgvnwrgsigigdvt--aavns---------------------qlsaisnav  234 (409)
                     +|+++|.+.+||.||++|+|++.++.+++|++|......+...  +....                      +.+.+....
T Consensus      310 li~~lD~~~~qV~Ie~~ivev~~~~~~~~~lGi~~~~~~~~~~~~~~~~~~~~~~~~~~~~~~~~~~~~~~~~~~~~~~~~~~~~~  389 (571)
T 6W6M_K         310 IIESLDIPVKQVQIEARIVTVKEGNLEELGVRWGVMSTNGSHSVGGSIESNLWQKGLLADDEFPVDEFLNVNLASTSANA  389 (571)
T ss_dssp            HHHHHCCCCCEEEEEEEEEEEEEEEEEEEEEEEEEEEECSSCEEESSHHHHHHTTTCCSCCSCCHHHHCSEEECCCSSSC
T ss_pred            HHHhcCCCCceEEEEEEEEEEEEcccchheeeeEeEeecCCCcccccccccccccccCCcCCCCCCcchhcccccccCcccc
Confidence           999999999999999999999999999999999998643221100  00000                     000000001


Q ORF11          235 STGGLTFV--SGGNLLDARLAAMEAHGKGKIISQPRVYTADREQARIGRGTEVPYQQSAGDGATSVSFKEASLALDVTPV  312 (409)
Q Consensus      235 stggltfv--sggnlldarlaameahgkgkiisqprvytadreqarigrgtevpyqqsagdgatsvsfkeaslaldvtpv  312 (409)
                     ++.++.++   .....+.+.|++|+++|.++++++|++++.||++|.|..|...|+++||......+.++.++.+.|+.|+|+|+
T Consensus      390 ~~~~~~~~~~~~~~~~~~~~l~~~l~ale~~g~~~~vls~P~v~t~ng~~A~i~~g~~~py~~~~~~~~~~~~~~~~~~~~gl~L~VtP~  469 (571)
```

```
T 6W6M_K          390 SSIAFQVAKLGSGTLLDLELSALQNESKAEIISSPRLITTNKQPAYIEQGTEIPYLESSSSGASTVAFKKAVLSLKVTPQ  469 (571)
T ss_dssp             EEEEEEEECSSSCCEEEEEEEEEEEEEEEEEEEEEEEEEEETTCCEEEEEEEEEEEESCCBSSSSBCCEEEEEEEEEEEEEE
T ss_pred             cceeeEeeecCCCccHHHHHHHHcccCCceeEecCEEEECCCceEEEEeCcEEeeEEeCCCCcceEEEEEeeEEEEEEeE
Confidence            11122222  124567888999999999999999999999999999999999998876665677889999999999999


Q ORF11           313 IDDTG-ALLSIKLSKDEPDYAS--AVDGVPPIKTASVTSRVRLKMGETVALGGVYSNAYGDQIKRVPGLSRLPWIGSWFQ  389 (409)
Q Consensus       313 iddtg-allsiklskdepdyas--avdgvppiktasvtsrvrlkmgetvalggvysnaygdqikrvpglsrlpwigswfq  389 (409)
                      |.+|+ |.|++.++.+......  +....|.+..+.++++++++||+|++|||+.+.......+++|+|++||+||+||+
T Consensus       470 i~~d~~I~l~i~~~~s~~~~~~~~~~~~~~~~p~i~~~~~~~t~v~v~~G~t~viGGl~~~~~~~~~~~~~vP~L~~IP~lg~lF~  549 (571)
T 6W6M_K          470 ITPDNRLVLDLSVTQDRRGETVKTGTGEAVSIDTQRIGTQVLVNNGETVVLGGIFQHSINNSVDKVPLLGDLPVLGALFR  549 (571)
T ss_dssp             EETTTEEEEEEEEEECEEEEEEEEBTEEEEEEEEEEEEEEEEEEETTCCEEEEEEEEEEEEEEEEEECTTGGGSTTGGGGSE
T ss_pred             ECCCCeEEEEEEEEecCCCceeeeCCCccceeeEEEEEEEEEeCCCCEEEEeEEEEEEeeeeeeccccccCccchHHhc
Confidence            99999 9999999988766542  2345799999999999999999999999999999999999999999999999999


Q ORF11           390 YRSHSDTNSELILFLTIRE  408 (409)
Q Consensus       390 yrshsdtnselilfltire  408 (409)
                      ++....+++|++|||||+|
T Consensus       550 ~~~~~~~~~~~elli~itp~I  568 (571)
T 6W6M_K          550 RTYEQMGKSELLIFVTPKV  568 (571)
T ss_dssp             EEEEEEEEEEEEEEEEEEE
T ss_pred             ceeeecccEEEEEEEeeEe
Confidence            9999999999999999997
```

```
ORF12, DB = PDB, Pfam, UniProt
no significant results
```

```
ORF13, DB = PDB, Pfam, UniProt
>1IRQ_A omega transcriptional repressor; transcriptional repressor, ribbon-helix-helix, GENE REGULATION; 1.5A
{Streptococcus pyogenes} SCOP: a.43.1.4
Probab=95.17  E-value=0.0026  Score=37.93  Aligned_cols=57  Identities=19%  Similarity=0.314  Sum_probs=43.3
Template_Neff=6.600

Q ORF13             6 NTPVRSAASEEKSVYLKCPGDLRDAFKAACASNDQNSSQVIRDFMRKYVAQHGQGRL   62 (63)
Q Consensus         6 ntpvrsaaseeksvylkcpgdlrdafkaacasndqnssqvirdfmrkyvaqhgqgrl   62 (63)
                      .+|.-+.......+-+.-|.+|...||..|+.++..-+.+|.+.++.|+..++.+.|
T Consensus        15 ~~~~~~~~k~~~~~~~tvrl~~elh~~lK~~A~~~g~sm~evi~eaI~~~l~~~~~~~~~~   71 (71)
T 1IRQ_A           15 DTPISAKKDIMGDKTVRVRADLHHIIKIETAKNGGNVKEVMDQALEEYIRKYLPDKL   71 (71)
T ss_dssp             ---------CCCEEEEEEEHHHHHHHHHHHHHHHTCCHHHHHHHHHHHHHHHHSTTCC
T ss_pred             CCCCccccCCccceEEEcCHHHHHHHHHHHHHhCCCHHHHHHHHHHHHHHHHHCCCCC
Confidence            455555555667777888999999999999999999999999999999999987654
```

Table S4. Python script used to extract coding sequences from GenBank files and saving them as Fasta (.faa) files.

```
from Bio import SeqIO

gb_file = input("Paste directory and file in format '/dir/file.extension'.\n")
gb_filename = gb_file
faa_filename = gb_filename.rsplit('.',1)[0] + '.faa'

if not gb_filename.endswith(('.gb', '.gbk')):
    print("Make sure your input is a genbank file. Accepted file extensions are '.gb' & '.gbk'")
else:
    orf = 1
    with open(gb_filename, "r") as input_handle, open(faa_filename, "w") as output_handle:
        for record in SeqIO.parse(gb_filename, "genbank"):
            for feature in record.features:
                if feature.type == "CDS":
                    assert len(feature.qualifiers['translation']) == 1
                    if "label" in feature.qualifiers:
                        output_handle.write(f'>{feature.qualifiers["label"][0]} '
                                            f'[{feature.qualifiers["product"][0]}] '
                                            f'[{record.name}]\n'
                                            f'{feature.qualifiers["translation"][0]}\n'
                                            )
                    else:
                        output_handle.write(f'>ORF{str(orf)} '
                                            f'[{feature.qualifiers["product"][0]}] '
                                            f'[{record.name}]\n'
                                            f'{feature.qualifiers["translation"][0]}\n'
                                            )
                        orf += 1
```
